# Effectiveness of Antiseizure Medication Duotherapies in Patients With Glioma: A Multicenter Observational Cohort Study

**DOI:** 10.1212/WNL.0000000000200807

**Published:** 2022-09-06

**Authors:** Pim B. van der Meer, Linda Dirven, Marta Fiocco, Maaike J. Vos, Mathilde C.M. Kouwenhoven, Martin J. van den Bent, Martin J.B. Taphoorn, Johan A.F. Koekkoek

**Affiliations:** From the Department of Neurology (P.M., L.D., M.J.B.T., J.A.F.K.), Leiden University Medical Center; Department of Neurology (L.D., M.J.V., M.J.B.T., J.A.F.K.), Haaglanden Medical Center, The Hague; Department of Biomedical Data Sciences (M.F.), Medical Statistics, Leiden University Medical Center; Mathematical Institute (M.F.), Leiden University; Department of Neurology (M.C.M.K.), Amsterdam University Medical Centers, location VUmc; and Brain Tumor Center at Erasmus Medical Center Cancer Institute (M.J.B.), Rotterdam, the Netherlands.

## Abstract

**Background and Objectives:**

About 30% of patients with glioma need an add-on antiseizure medication (ASM) due to uncontrolled seizures on ASM monotherapy. This study aimed to determine whether levetiracetam combined with valproic acid (LEV + VPA), a commonly prescribed duotherapy, is more effective than other duotherapy combinations including either LEV or VPA in patients with glioma.

**Methods:**

In this multicenter retrospective observational cohort study, treatment failure (i.e., replacement by, addition of, or withdrawal of an ASM) for any reason was the primary outcome. Secondary outcomes included (1) treatment failure due to uncontrolled seizures and (2) treatment failure due to adverse effects. Time to treatment failure was estimated from the moment of ASM duotherapy initiation. Multivariable cause-specific Cox proportional hazard models were estimated to study the association between risk factors and treatment failure. The maximum duration of follow-up was 36 months.

**Results:**

A total of 1,435 patients were treated with first-line monotherapy LEV or VPA, of which 355 patients received ASM duotherapy after they had treatment failure due to uncontrolled seizures on monotherapy. LEV + VPA was prescribed in 66% (236/355) and other ASM duotherapy combinations including LEV or VPA in 34% (119/355) of patients. Patients using other duotherapy vs LEV + VPA had a higher risk of treatment failure for any reason (cause-specific adjusted hazard ratio [aHR] 1.50 [95% CI 1.07–2.12], *p* = 0.020), due to uncontrolled seizures (cause-specific aHR 1.73 [95% CI 1.10–2.73], *p* = 0.018), but not due to adverse effects (cause-specific aHR 0.88 [95% CI 0.47–1.67], *p* = 0.703).

**Discussion:**

This observational cohort study suggests that LEV + VPA has better efficacy than other ASM combinations. Similar toxicities were experienced in the 2 groups.

**Classification of Evidence:**

This study provides Class III evidence that for patients with glioma with uncontrolled seizures on ASM monotherapy, LEV + VPA has better efficacy than other ASM combinations.

Seizures occur frequently in patients with glioma, with the preoperative seizure incidence in diffuse gliomas ranging from ∼25% in World Health Organization (WHO) grade 4 glioblastoma isocitrate dehydrogenase (IDH)-wildtype to ∼75% in grade 2 diffuse astrocytoma IDH-mutant and oligodendroglioma IDH-mutant 1p/19q codeleted patients.^1^ Antiseizure medications (ASMs) are the cornerstone of anticonvulsant treatment, with levetiracetam (LEV) and valproic acid (VPA) being the most commonly prescribed ASMs in the glioma population.^2-4^ Recently, it has been shown that first-line monotherapy with LEV has favorable efficacy over VPA, while having a similar level of toxicity.^5^ In about 30% of patients with glioma, seizures are inadequately controlled by ASM monotherapy, and these patients generally need ASM polytherapy treatment.^5^ Preclinical evidence showed that especially the combination of LEV + VPA leads to a strong enhancement of anticonvulsant effects across different preclinical models and stood out compared with other ASM combinations, suggesting a beneficial synergistic effect (i.e., an interaction effect between 2 drugs, resulting in a joint effect that is greater than the sum of the individual effects of each drug).^6^ Among LEV's mechanism of action is the modulation of synaptic neurotransmitter release through binding to the synaptic vesicle glycoprotein 2A in the brain,^7^ indirectly affecting GABAergic neurotransmission as well.^8^ VPA is regarded as having multiple mechanisms of action, including GABAergic potentiation, glutamate inhibition, and blockade of voltage-dependent sodium currents.^6,9^

The International League Against Epilepsy recommends to use either an efficacy or effectiveness outcome as the primary outcome in comparative ASM studies.^10^ The efficacy of an ASM means its ability to achieve seizure freedom, while effectiveness includes both efficacy and tolerability, of which the latter encompasses the incidence, severity, and impact of ASM-related adverse effects, which is most importantly reflected in ASM discontinuation due to intolerable or life-threatening adverse reactions.^11^ In 2 studies conducted in a large neuro-oncology outpatient clinic in the Netherlands, LEV + VPA was the most frequently prescribed and the most efficacious polytherapy combination in patients with brain tumor.^12,13^ However, methodologic issues such as the competing risk of death were not taken into account, hampering reliable interpretation of results.^11^ Therefore, we investigated whether LEV + VPA had better effectiveness, efficacy, and/or tolerability compared with other ASM duotherapy combinations, including LEV or VPA, in patients with glioma with uncontrolled seizures on first-line monotherapy, specifically by estimating time to ASM treatment failure for any reason, due to uncontrolled seizures, and due to adverse effects.

## Methods

### Study Population and Procedures

Details about the study cohort and methods have already been published elsewhere.^5^ In short, the study population consisted of consecutive patients with a histologically diagnosed grade 2–4 glioma ([anaplastic] astrocytoma, [anaplastic] oligoastrocytoma, [anaplastic] oligodendroglioma, or glioblastoma) according to the WHO 2016 guidelines,^14^ following biopsy or surgical (re)resection in Haaglanden Medical Center, The Hague, Amsterdam University Medical Centers, or Erasmus Medical Center Rotterdam, between January 1, 2004, and January 1, 2018. In the original cohort, we included patients with epilepsy who received first-line monotherapy treatment with LEV or VPA. Regarding the current study, patients who showed treatment failure on either first-line LEV or VPA monotherapy due to uncontrolled seizures were included. Patients who (1) were prescribed an add-on for a predetermined maximum term, (2) showed treatment failure on their first-line LEV or VPA due to other reasons than uncontrolled seizures, and (3) showed treatment failure due to uncontrolled seizures but were treated with another ASM as monotherapy were excluded. Incrementing the ASM dose and deciding whether the addition of an ASM due to uncontrolled seizures was warranted were according to the judgement of the treating physician, taking into account the maximum daily dose according to national guidelines. This resulted in 2 groups that were compared: LEV + VPA vs other ASM duotherapy including either LEV or VPA.

Patients' charts were examined to extract baseline sociodemographic and clinical characteristics, including radiologic response data (i.e., tumor progression) according to the Response Assessment in Neuro-Oncology criteria.^15^ For this study, baseline was defined as the starting date of ASM duotherapy initiation. Although LEV and VPA have equal defined daily dosages (DDDs), this is not true for many other ASMs. Therefore, the ASM load was calculated for each patient, defined as the sum of the ratio between the prescribed daily dosage and the DDD of each individual ASM included in the ASM treatment combination (eTable 1, links.lww.com/WNL/C119).^16^

### Outcomes

The primary outcome was time to treatment failure for any reason from the initiation of ASM duotherapy, which is a measure for the effectiveness of ASM treatment and encompasses both ASM efficacy and tolerability.^17^ Treatment failure was defined as withdrawal, replacement, or the addition of an ASM. The following conditions were not considered treatment failure: a change in the dosage of the initial ASM combination, addition of an ASM taken only as needed, addition of an ASM with an indication other than seizures, use of a temporary prophylactic ASM as an add-on during a perioperative period, poor adherence less than 1 week, or replacement with a nonoral ASM in the end-of-life phase due to swallowing difficulties. Secondary outcomes included (1) time to treatment failure due to uncontrolled seizures from ASM duotherapy initiation, as a measure of efficacy; (2) time to treatment failure due to adverse effects from ASM duotherapy initiation, as a measure of tolerability; (3) time to recurrent epileptic seizure from ASM duotherapy initiation, which is a measure for efficacy; and (4) level of toxicity, defined as severity (grade 1–5) of intolerable adverse effects leading to ASM discontinuation according to the Common Terminology Criteria for Adverse Events version 5.0,^18^ as a measure of tolerability. To determine how likely the intolerable adverse effect was attributable to the ASM combination, it was evaluated whether the adverse effects improved or not, typically in a period of 1–2 months. If the adverse effects improved after ASM discontinuation, the adverse effects were considered attributable to the ASM combination^19^; if not, this seemed less likely. The maximum duration of follow-up was 36 months. Patients were censored if they had not shown the event of interest and were still alive at 36 months or were lost to follow-up.

### Statistics

Time to treatment failure and time to recurrent seizure of 2 ASM duotherapy treatment groups (LEV + VPA vs other duotherapy including LEV or VPA) were compared, from the moment of ASM duotherapy initiation. Multivariable Cox proportional hazard models were estimated to study the association between risk factor (i.e., ASM duotherapy) and treatment failure. In case of an etiologic research question (in contrast to prediction research) and the presence of competing risks, a Cox proportional hazards model is preferred, and potential confounders should be selected on preexisting knowledge.^11,20^ Four different competing risk models were estimated: (1) treatment failure for any reason (event of interest) vs death, (2) treatment failure due to uncontrolled seizures (event of interest) vs treatment failure due to other reasons than uncontrolled seizures and death, (3) treatment failure due to adverse effects (event of interest) vs treatment failure due to other reasons than adverse effects and death, and (4) recurrent seizure (event of interest) vs treatment failure before a recurrent seizure has occurred and death.^21^ All Cox models were repeated for subgroup analyses within the other duotherapy group with the same potential confounders, and the ASM combinations with the LEV group were compared with the VPA group. The proportional hazards assumption was checked by considering Schoenfeld residuals, nonlinearity by Martingale residuals, and influential observations by deviance residuals. The censoring distribution was checked by modeling time to censoring in the same way as time to any event of interest. In the censoring model, the event of interest was censoring. Therefore, patients who were lost to follow-up had an “event.” Patients who were not censored (i.e., those who experienced the original event of interest) were now considered censored because their censoring time was not observed. In our study, all baseline covariates, which were significant for the time to the event of interest, were included in the model. To assess the difference between the cumulative incidences, the Gray test was used.^22^ Presence of radiologic tumor progression at the time of treatment failure due to uncontrolled seizures, presence of residual tumor at baseline, severity of intolerable adverse effects (grade 1/2 vs 3/4) in patients using LEV + VPA vs other ASM duotherapy, and whether or not adverse effects improved were compared using the χ^2^ test, whereas dosage at the moment of treatment failure was analyzed using the independent *t* test. The following potential confounding variables were considered in each analysis and were considered to be relevant for the outcome based on the previous literature and expert opinion: age, sex, tumor grade, IDH-mutation status, surgical resection, radiotherapy, systemic therapy, tumor location, Karnofsky Performance Status, history of a psychiatric disorder (depression, anxiety, or psychotic disorder), and seizure type. The median follow-up time (including interquartile range [IQR]) was calculated with the reverse Kaplan-Meier methodology. Statistical analyses were performed using statistical packages SPSS version 25.0 and R, an open software environment.^23,24^ All analyses concerning the competing risk models were performed in R with the cmprsk library.^21^ A *p* value of <0.05 was considered statistically significant.

### Standard Protocol Approvals, Registrations, and Patient Consents

The medical ethics committee of each institution approved the protocol, and the consent of patients was obtained according to the institutions' policy.

### Data Availability

Data are available on reasonable request.

## Results

### Patient Characteristics

Baseline cohort characteristics of patients on ASM duotherapy are reported in Table 1. The original study population consisted of 1,435 patients prescribed first-line monotherapy LEV or VPA. A total of 382 unique patients experienced treatment failure due to uncontrolled seizures on their first-line ASM, of which 7% (27/382) started with another ASM as monotherapy and 93% (355/382) of patients started with ASM duotherapy (Figure 1). LEV + VPA was prescribed to 236 (236/355 = 66%) and other ASM duotherapy, including LEV or VPA, to 119 (119/355 = 34%) patients (Table 2). Other ASM duotherapy consisted of 15 unique combinations, of which 68 patients used a combination with VPA and 51 with LEV. LEV + clobazam (19/119 = 16%) and VPA + phenytoin (18/119 = 15%) were prescribed most commonly as other ASM duotherapy. At baseline, 62% (147/236) of patients in the LEV + VPA group had received surgical resection and 49% (58/119) of patients in the other ASM duotherapy group. The presence of residual tumor did not differ significantly between LEV + VPA and other ASM duotherapy (73% [108/147] vs 79% [46/58] subtotal resection, *p* = 0.384). The median follow-up time was 16 months (IQR 5–36 months).

**Table 1:**
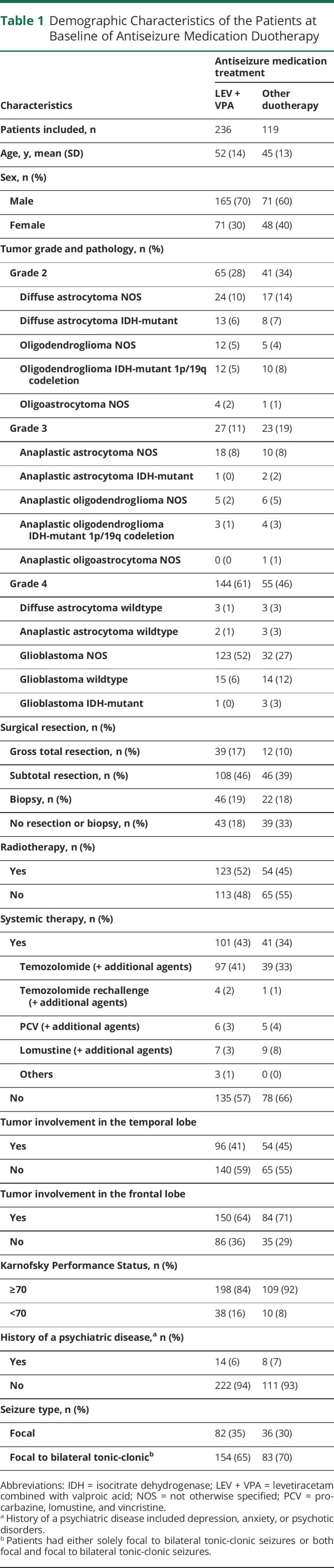
Demographic Characteristics of the Patients at Baseline of Antiseizure Medication Duotherapy

**Figure:**
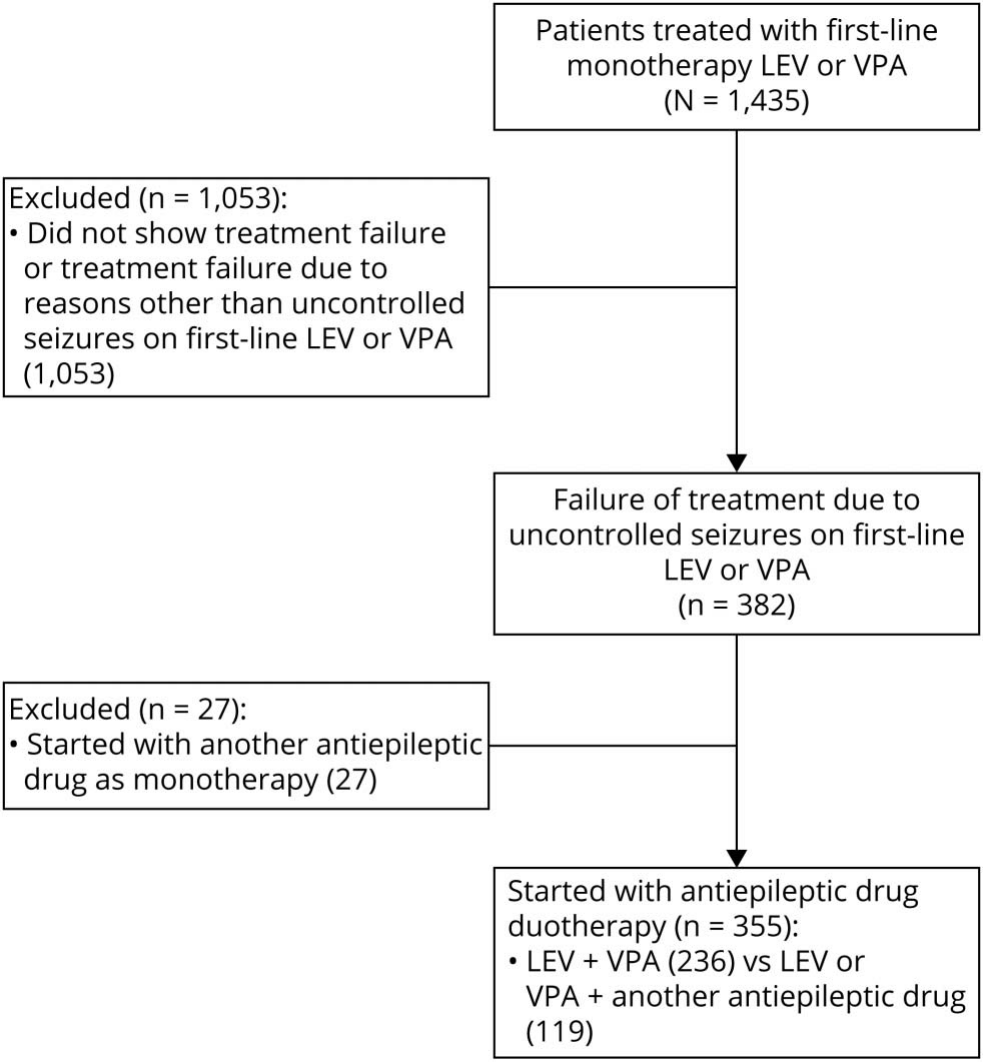
Flow Diagram of Included and Excluded Patients LEV = levetiracetam; VPA = valproic acid.

**Table 2:**
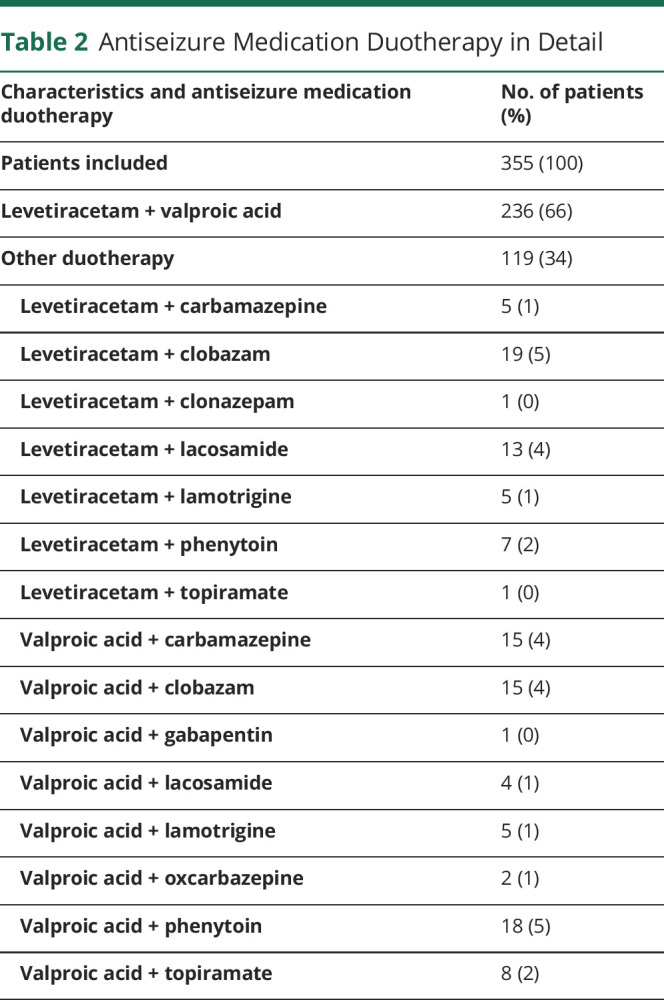
Antiseizure Medication Duotherapy in Detail

### Time to Treatment Failure

A total of 42% (99/236) of patients who used the combination of LEV + VPA showed treatment failure within 36 months of follow-up vs 55% (65/119) of patients who used ASM duotherapy with either LEV or VPA combined with another ASM. The main reason of treatment failure for LEV + VPA and other ASM duotherapy was uncontrolled seizures (23% [55/236] vs 31% [37/119] of patients), followed by adverse effects (15% [35/236] vs 13% [15/119] of patients), other reasons (3% [7/236] vs 7% [8/119] of patients), and withdrawal due to remission of seizures (1% [2/236] vs 4% [5/119] of patients). The cumulative incidence of treatment failure for any reason of LEV + VPA and other ASM duotherapy at 12 months was 37% (95% CI 30%–43%) vs 50% (95% CI 40%–59%; eTable 2, links.lww.com/WNL/C119). The cumulative incidence of LEV + VPA vs other ASM duotherapy at 12 months for treatment failure due to uncontrolled seizures was 21% (95% CI 16–26) vs 29% (95% CI 21%–38%) and for treatment failure due to adverse effects was 13% (95% CI 9%–18%) vs 11% (95% CI 6%–18%), respectively (eTable 3). Other ASM duotherapy had a significantly higher risk of treatment failure for any reason compared with the combination of LEV + VPA (cause-specific adjusted hazard ratio [aHR] 1.50 [95% CI 1.07–2.12], *p* = 0.020; Table 3). With regard to specific reasons of treatment failure, patients using other ASM duotherapy were more likely to experience treatment failure due to uncontrolled seizures (cause-specific aHR 1.73 [95% CI 1.10–2.73], *p* = 0.018; eTable 4), but not treatment failure due to adverse effects (cause-specific aHR 0.88 [95% CI 0.47–1.67], *p* = 0.703; eTable 5).

**Table 3:**
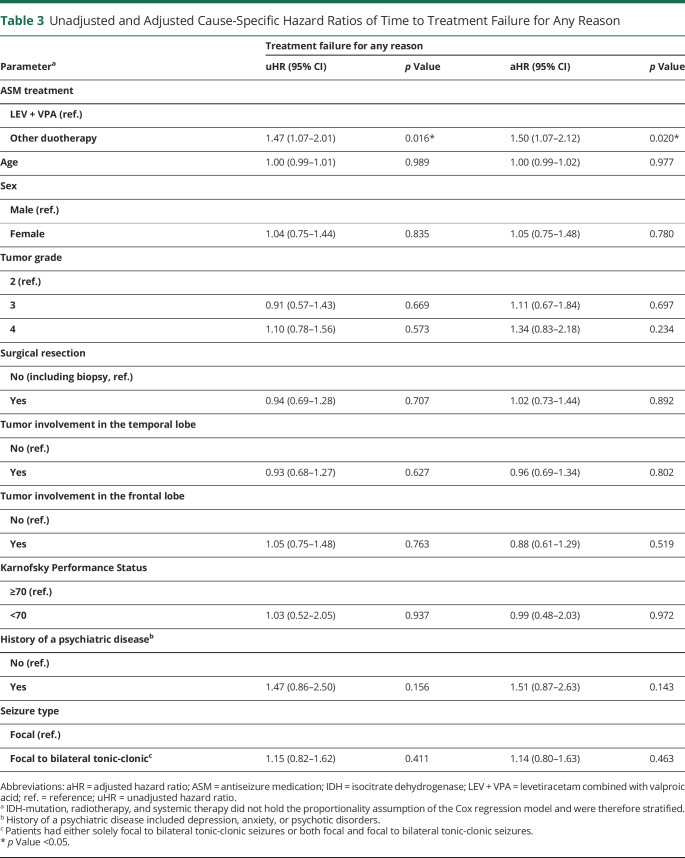
Unadjusted and Adjusted Cause-Specific Hazard Ratios of Time to Treatment Failure for Any Reason

The mean ASM load at the moment of treatment failure in patients on LEV + VPA and other ASM duotherapy was similar for those showing treatment failure due to uncontrolled seizures (2.44 [SD = 0.58] vs 2.30 [SD = 0.61] ASM load, *p* = 0.276) or intolerable adverse effects (2.03 [SD = 0.46] vs 1.84 [SD = 0.39] ASM load, *p* = 0.215). Tumor progression at the time of treatment failure due to uncontrolled seizures did not differ significantly between LEV + VPA and other ASM duotherapy (45% [25/55] vs 38% [14/37], *p* = 0.469).

### Time to Recurrent Seizure

A recurrent seizure within 36 months of follow-up occurred in 78% (182/232) of patients on LEV + VPA vs 85% (99/116) on other ASM duotherapy combinations. The cumulative incidence of recurrent seizure at 12 months was 74% (95% CI 68%–79%) for LEV + VPA and 87% (95% CI 79%–92%) for other ASM duotherapies (eTable 6, links.lww.com/WNL/C119). Patients in the other ASM duotherapy group had a significantly higher risk of having a recurrent seizure (cause-specific aHR 1.66 [95% CI 1.28–2.17], *p* < 0.001; Table 4).

**Table 4:**
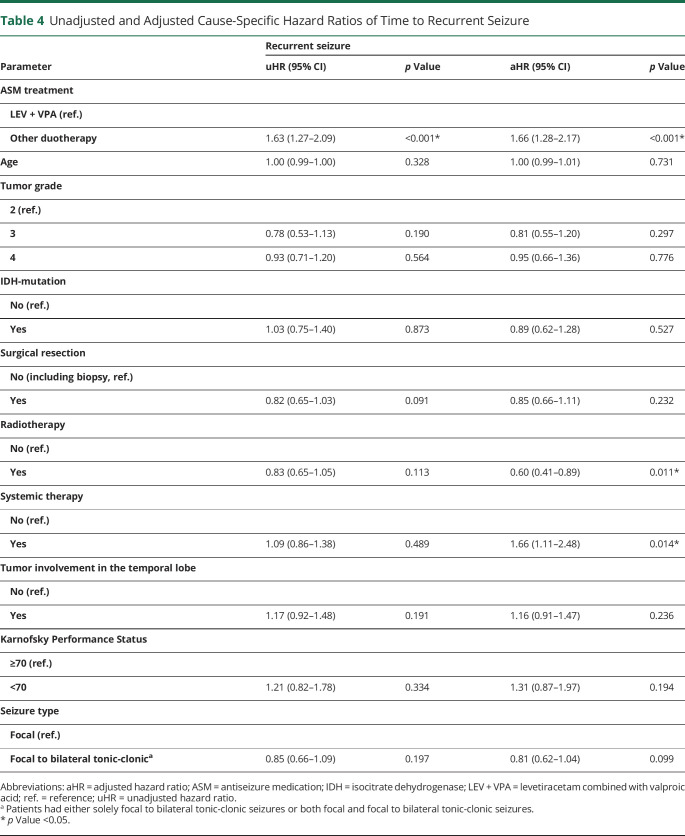
Unadjusted and Adjusted Cause-Specific Hazard Ratios of Time to Recurrent Seizure

### Intolerable Adverse Effects Leading to Treatment Failure

A total of 47 adverse effects were reported in the LEV + VPA group that led to treatment failure, occurring in 15% (35/236) of patients. Similarly, 24 adverse effects leading to treatment failure were reported in 13% (15/119) of patients in the other ASM duotherapy group (Table 5). Hepatobiliary disorders occurred only in the LEV + VPA group (2/47 = 4%) and were transient in half of these cases (1/2 = 50%). Psychiatric disorders occurred in 17% (8/47) of patients in the LEV + VPA group and improved in almost all (7/8 = 88%), whereas psychiatric disorders occurred in 8% (2/24) of patients in the other ASM duotherapy group and improved in none (0/2 = 0%). The 2 most common intolerable adverse effects for the combination of LEV + VPA were tremor (8/47 = 17%) and decreased platelet count (4/47 = 9%), and for the other ASM duotherapies, this was somnolence (3/24 = 13%) and rash (3/24 = 13%; eTable 7, links.lww.com/WNL/C119). Only a small number of adverse effects in the LEV + VPA group and the other ASM duotherapy group were grade 3 or 4 (23% [11/47] vs 21% [5/24], *p* = 0.389) or did not improve after discontinuation of an ASM (11% [5/47] vs 21% [5/24], *p* = 0.464).

**Table 5:**
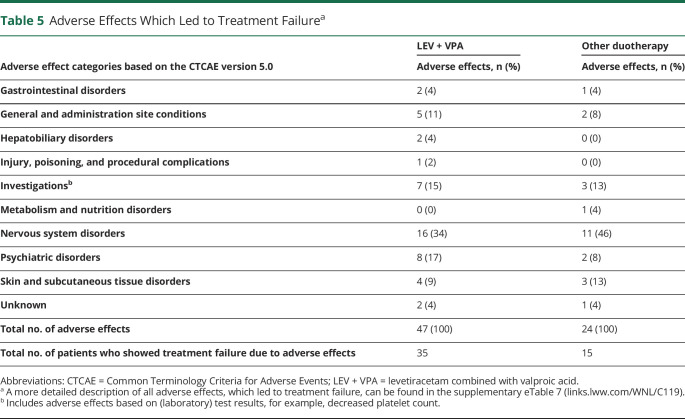
Adverse Effects Which Led to Treatment Failure^a^

### Subgroup Analyses Within the Other Duotherapy Group

Within the other ASM duotherapy group, 68 patients were on a combination with VPA and 51 patients on a combination with LEV. Treatment failure rates for the combinations with VPA and the combinations with LEV were as follows: treatment failure for any reason was 57% (39/68) vs 51% (26/51), treatment failure due to uncontrolled seizures was 27% (19/68) vs 35% (18/51), and treatment failure due to adverse effects was 21% (14/68) vs 2% (1/51), respectively. The percentages of a recurrent seizure for patients on combinations with VPA vs combinations with LEV were 85% (55/65) vs 86% (44/51). There was no significant difference in the risk of having treatment failure for any reason when combinations with VPA were compared with combinations with LEV (cause-specific aHR 1.32 [95% CI 0.75–2.31], *p* = 0.331), treatment failure due to uncontrolled seizures (cause-specific aHR 1.15 [95% CI 0.56–2.37], *p* = 0.698), or a recurrent seizure (cause-specific aHR 0.99 [95% CI 0.65–1.51], *p* = 0.968). However, patients on combinations with VPA had a significantly higher risk of experiencing treatment failure due to adverse effects (cause-specific aHR 10.10 [95% CI 1.31–78.04], *p* = 0.027; data not shown).

### Classification of Evidence

This study provides Class III evidence that for patients with glioma with uncontrolled seizures on ASM monotherapy, LEV + VPA has better efficacy than other ASM combinations.

## Discussion

The aim of this retrospective observational cohort study was to evaluate the effectiveness of combined LEV + VPA compared with other ASM duotherapy combinations including either LEV or VPA. We found that LEV + VPA has a similar level of toxicity compared with other duotherapies, but better efficacy. Greater efficacy of LEV + VPA was supported by a lower risk both for treatment failure due to uncontrolled seizures and a recurrent seizure. The ASM load at the moment of treatment failure was similar between the 2 groups, which suggests that the difference in efficacy between the 2 groups of duotherapy is not explained by a discrepancy in dose escalation. This study showed as well that 1-year seizure freedom directly after ASM duotherapy initiation is uncommon because the cumulative incidence of a recurrent seizure at 12 months was equal to 74% (95% CI 68%–79%) and 87% (95% CI 79%–92%) for the combination of LEV + VPA and other ASM duotherapies, respectively. Although polytherapy is generally considered as posing a higher risk for adverse effects,^25^ this was not shown in our study because the cumulative incidence of treatment failure due to adverse effects at 6 months was equal to 10%–11%, compared with 11%–12% at 6 months in first-line monotherapy ASM treatment in patients with glioma,^5^ and 10%–14% in patients with non–brain tumor-related epilepsy at 6 months.^26,27^ Of interest, other ASM duotherapy combinations with LEV were tolerated very well compared with combinations with VPA, given only 2% (1/51) of the combinations with LEV showed treatment failure due to adverse effects. This implies that if patients on LEV + VPA experience intolerable adverse effects ascribed to VPA, replacement of VPA by another ASM will probably be tolerated well.

First-line LEV has shown superior efficacy compared with VPA in the glioma population, with a similar level of toxicity, and should be considered as the preferred first-line ASM in this population.^5^ If patients fail to respond adequately to first-line LEV and need an add-on ASM, VPA appears to be the preferred choice. Valproic acid may lead to hematologic toxicity, such as decreased platelet count, platelet dysfunction, and coagulation abnormalities. This particularly represents a concern for patients with glioma who are on chemotherapy or in whom a surgical intervention is intended.^28^ Because all the other 15 unique ASM duotherapy combinations with LEV and VPA were used by a limited number of patients, we could not draw conclusions about other possible synergistic effects. It is noteworthy that the duotherapy combination of LEV with lacosamide has shown synergistic effects in preclinical studies and high efficacy in patients with brain tumor.^29,30^ Still, about a quarter of patients on duotherapy show treatment failure due to uncontrolled seizures and need a third ASM. Whether LEV + VPA is truly the most efficacious combination in patients with glioma cannot be derived with certainty from our study. However, with a total number of up to 20 ASMs, ∼200 possible duotherapy combinations can be made; it is extremely difficult to discover the most effective duotherapy combination.^31^ Despite the general recommendation that polytherapy should only be considered when 2 attempts to monotherapy with ASMs have not resulted in seizure freedom,^32^ 2 subsequent trials of monotherapy were found to be uncommon in our cohort (only 7% in this study). One of the most important reasons for the recommendation of a subsequent monotherapy trial instead of polytherapy is that ASM monotherapy treatment is associated with fewer adverse effects in patients with epilepsy.^33^ However, this has not been substantiated in the glioma population, and given the suggested beneficial synergistic effect of the combination of LEV + VPA in patients with glioma, reflected in a better efficacy, initiation of polytherapy if first-line monotherapy treatment fails seems to be an adequate treatment strategy in patients with glioma. We believe that our results have high external validity and can be generalized to other neuro-oncology clinics internationally.

Considering that patients may metabolize ASMs differently based on their pharmacogenetics, serum levels could have been a more reliable estimate than mean ASM load. However, information on ASM serum levels was not available because they were rarely monitored by neuro-oncology professionals in clinical practice during follow-up.^34^ In our analyses, we only have adjusted for psychiatric comorbidities, while potentially other comorbidities may have contributed to treatment failure. Although valproic acid does not have any drug-drug interactions with levetiracetam, it does have interactions with other ASMs, such as phenytoin,^35^ which might have contributed to treatment failure in the other ASM duotherapy group. Only a third of patients with glioma need ASM duotherapy. This, in combination with the relative rarity of the disease, hinders to include a great number of patients, therefore limiting statistical power in this study.

The inclusion of 236 patients on a specific duotherapy combination in such relatively rare disease as diffuse glioma can be called unique, given the high number of possible duotherapy combinations. Currently, there is a lack of randomized controlled trials (RCTs) and/or well-conducted observational studies on ASM duotherapies in patients with glioma. In our view, the results of this manuscript are clinically very relevant and can help guide clinicians in their choice for ASM duotherapy treatment. The results of our study are in line with previous data, which showed that the combination of LEV + VPA had a more favorable antiseizure effect compared with other ASM duotherapy combinations. Only 40%–41% of patients on LEV + VPA were not seizure-free in the 2 previous studies,^12,13^ while at 12 months the cumulative incidence for a recurrent seizure was 74% for patients on LEV + VPA in our study. However, methodologic issues were not taken into account, such as competing risks,^11^ hampering adequate interpretation. In addition, both previous studies did not specifically define seizure freedom, that is, it was unclear how long patients had to be free of seizures in order to be regarded as seizure-free.^12,13^ We believe that our results provide a reliable estimate of the risk for having a recurrent seizure if a patient starts with the combination of LEV + VPA.

To conclude, this retrospective observational cohort study suggests that LEV + VPA is more effective than other ASM duotherapy combinations with either LEV or VPA, while the level of toxicity is similar. Duotherapy with LEV + VPA seems an appropriate choice in patients with glioma if seizures are not adequately under control with ASM monotherapy LEV or VPA.

## Glossary

aHR: adjusted hazard ratio
ASM: antiseizure medication
DDD: defined daily dosage
IDH: isocitrate dehydrogenase
IQR: interquartile range
LEV: levetiracetam
VPA: valproic acid
WHO: World Health Organization
